# Pilot study on the value of echocardiography combined with lung ultrasound to evaluate COVID-19 pneumonia

**DOI:** 10.1186/s12947-021-00271-0

**Published:** 2022-01-19

**Authors:** Jing Han, Xi Yang, Wei Xu, Ronghua Jin, Weiyuan Liu, Lei Ding, Sha Meng, Yuan Zhang, Jin Li, Ying Zheng, Haowen Li, Fankun Meng

**Affiliations:** 1 Ultrasound and Functional Diagnosis Center, Beijing You An Hospital, Capital Medical University, Beijing, 100069 China; 2 Department of ultrasound, Hanyang Hospital Affiliated to Wuhan University of science and technology, Wuhan, 430050 China; 3 Key Laboratory of Carcinogenesis and Translational Research (Ministry of Education/Beijing), Department of Hepato-Pancreato-Biliary Surgery, Peking University Cancer Hospital & Institute, Beijing, 100142 China; 4 Beijing You An Hospital, Capital Medical University, Beijing, 100069 China; 5 Department of Science and Technology Department, Beijing You An Hospital, Capital Medical University, Beijing, 100069 China; 6 Department of Ultrasound, Beijing Hospital of Chinese Traditional and Western Medicine, Beijing, 100069 China; 7 Ultrasonography, China Aerospace Science & Industry Corporation 731 Hospital, Beijing, 100074 China

**Keywords:** Echocardiography, Lung ultrasound score, COVID-19, Pulmonary arterial pressure

## Abstract

**Background:**

This study aimed to investigate the relationship between echocardiography results and lung ultrasound score (LUS) in coronavirus disease 2019 (COVID-19) pneumonia patients and evaluate the impact of the combined application of these techniques in the evaluation of COVID-19 pneumonia.

**Methods:**

Hospitalized COVID-19 pneumonia patients who underwent daily lung ultrasound and echocardiography were included in this study. Patients with tricuspid regurgitation within three days of admission were enrolled. Moreover, the correlation and differences between their pulmonary artery pressure (PAP) and LUS on days 3, 8, and 13 were analyzed. The inner diameter of the pulmonary artery root as well as the size of the atria and ventricles were also considered.

**Results:**

The PAP on days 3, 8, and 13 of hospitalization was positively correlated with the LUS (*r* = 0.448, *p* = 0.003; *r* = 0.738, *p* < 0.001; *r* = 0.325, *p* = 0.036, respectively). On day 8, the values of both PAP and LUS were higher than on days 3 and 13 (*p* < 0.01). Similarly, PAP and LUS were significantly increased in 92.9% (39/42) and 90.5% (38/42) of patients, respectively, and at least one of these two values was positive in 97.6% (41/42) of cases. The inner diameters of the right atrium, right ventricle, and pulmonary artery also differed significantly from their corresponding values on days 3 and 13 (*p* < 0.05).

**Conclusions:**

PAP is positively correlated with LUS in COVID-19 pneumonia. The two values could be combined for a more precise assessment of disease progression and recovery status.

## Background

On February 11, 2020, the World Health Organization (WHO) declared the emergence of coronavirus disease 2019 (COVID-19) caused by severe acute respiratory syndrome (SARS) coronavirus-2 in Wuhan, Hubei, China [1]. With the rapid worldwide spread of COVID-19 [2, 3], real-time reverse-transcriptase polymerase chain reaction (RT-PCR) and high-resolution chest computed tomography (CT) have become progressively insufficient to meet diagnostic demands. Several countries and regions have gradually adopted ultrasonography as the basic examination method [4–6], especially Italy [7]; hence, improvements in the ultrasound diagnostic value for COVID-19 pneumonia are required. At present, some reports in the literature [8, 9] describe the combination of pulmonary and cardiac ultrasound to evaluate patients with COVID-19 pneumonia. However, the correlation between pulmonary arterial pressure and lung ultrasound score (LUS) has not been investigated in detail. If these two values are related, the results of one of these investigations may reflect the results of the other, thereby reducing the burden of work in a specific environment. The present study aimed to examine the correlations between these values for the progression evaluation and treatment monitoring of patients with COVID-19 pneumonia.

## Methods

### Study design

This study was supported by the Ministry of Science and Technology of the People’s Republic of China (grant number: 2020YFC0844900). The study was approved by the Ethics Committee of Beijing You An Hospital, affiliated with the Capital Medical University ([2020]020). Informed consent was obtained from all patients included in the study. In accordance with the WHO guidelines for COVID-19 spread prevention, our hospital’s infection prevention and control procedures were strictly implemented, ensuring that none of the health workers involved in the study were infected with the disease.

The present study included hospitalized COVID-19 pneumonia patients (with diagnosis confirmed by RT-PCR and CT) who underwent dynamic lung ultrasound and echocardiographic observation upon hospital admission. The combined use of the two modalities for evaluating lung lesions was assessed, and the correlations between the data provided were calculated.

A total of 138 patients with COVID-19 pneumonia were treated at our hospital. According to the Chinese government policy, these patients must be hospitalized for observation and treatment upon diagnosis confirmation; therefore, the hospitalization rate of patients with COVID-19 pneumonia is 100%. Among this group, we selected patients with tricuspid valve regurgitation. Since the proportion of patients with this disorder increased by the third day of hospitalization, we included patients with evident tricuspid regurgitation within 3 days of admission (42 cases). Afterward, LUS assessment and echocardiographic examinations were conducted daily. Other studies, including our previous research, suggest that the interval between the appearance of symptoms and the most severe stage of the disease is approximately 10 days [10, 11], and hospitalization is required approximately 2 days after the onset of the first symptoms. The mean hospital stay duration was 15 days. Accordingly, days 3, 8, and 13 were selected to compare LUS, pulmonary arterial pressures, and atrial and ventricular diameters. General symptoms, including fever, cough, and dyspnea, were recorded, as well as the history of underlying heart and lung disease. The sample size of this study was determined using R [R Core Team (2019), Version 3.6.1] with RStudio (Version 1.2.5019) and Pwr Package prior to commencing the experiment with a view of obtaining a power of 80%. Calculation was based on the lines of Cohen (1988) using in particular the same notations for effect sizes [12]. The estimated sample size needed to reach 80% power on the 0.05 significance level (two-sided test) with a correlation coefficient (r) smaller than 0.5, leaded to a sample size of 28 cases.

### Lung ultrasound

**Fig. 1:**
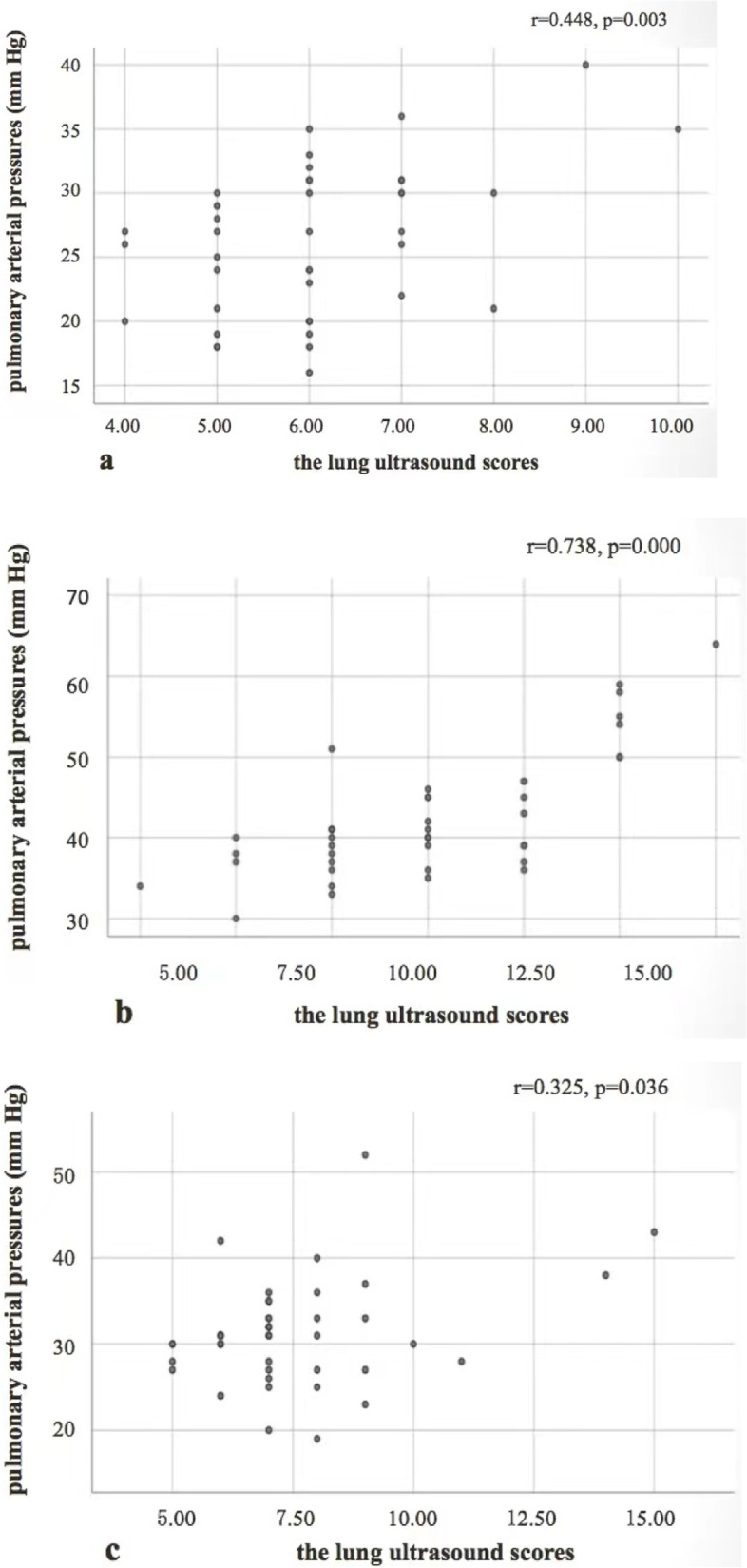
Correlation between LUS and pulmonary arterial pressure in 42 patients with COVID-19 pneumonia: (**a**) day 3; (**b**) day 8; (**c**) day 13

**Fig. 2:**
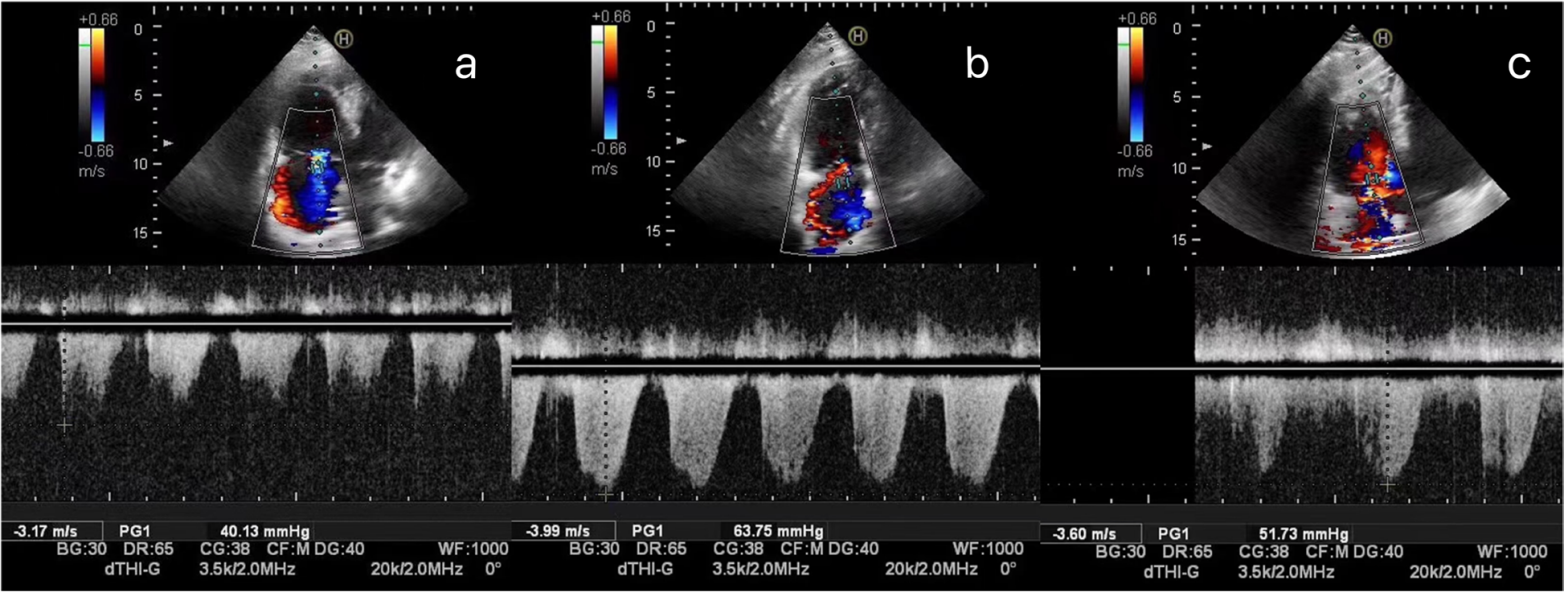
Parameters of a 60-year-old male patient with pulmonary hypertension, presenting with tricuspid regurgitation on color Doppler echocardiography. The systolic pulmonary artery pressure was estimated on days 3 (**a**), 8 (**b**), and 13 (**c**), using the gradient of tricuspid regurgitation pressure. The patient remained on extracorporeal membrane oxygenation support and exhibited low fever with stable heart rate and breathing

Rouby [13] proposed to divide one lung into six zones each for examination. In the present study, the lungs were both divided into three areas, with the anterior and posterior axillary lines as the boundaries: anterior, middle, and posterior (Fig. 1). Each area was further divided into superior and inferior regions. The results were recorded as the following four basic types (Fig. 2): type N: ≤ 2 separated B-lines, indicating good lung inflation; type B1: multiple B-lines, separated by approximately 7 mm (B7 lines); type B2: multiple B-lines, separated by ≤3 mm (B3 lines); and type C: hepatization or fragmentation, with dynamic bronchial inflation sign, and with or without pleural effusion, indicating lung consolidation. The LUS was based on the above four types: *N* = 0, B1 = 1, B2 = 2, and C = 3. In each region, the most severe sign was considered the final score. All patients underwent lung ultrasonographic examination, and the sum of the scores of the 12 regions was recorded. All lung ultrasound scans were performed by two experienced sonologists.

### Echocardiography

All the patients underwent echocardiographic examinations daily. Routine parasternal, apical, and other cardiac section scans were performed. The European Society of Cardiology (ESC) and the European Respiratory Society (ERS) indicated the guidelines for diagnosing pulmonary hypertension (PH). The screening criteria for PH are mainly based on the tricuspid regurgitation peak velocity and systolic pulmonary artery pressure [14, 15]. Left atrial volume (LAV), left ventricular volume, and right atrial volume (RAV) were estimated using the disk summation algorithm (Simpson’s technique) following a biplanar approach from the four apical chambers [16, 17]. The pulmonary artery (PA) root diameter, right ventricular base diameter, and right ventricular internal dimension were recorded.

### Statistical analysis

The sample size (42 > 28cases) was deemed sufficient for this study. Continuous data were expressed as mean ± standard deviation. Non-parametric tests were used to assess non-normally distributed data. The Mann–Whitney U test was used to compare two independent samples, and the Kruskal–Wallis test for multiple independent samples. Pearson’s correlational analysis was performed to assess the correlations between LUS and pulmonary artery pressure. The analysis of variance was used to analyze the differential distribution of data between more than two groups (LAV), with subsequent post hoc correction for multiple comparisons. A *p-*value < 0.05 was deemed statistically significant. The statistical software SPSS 26.0 (IBM, Armonk, NY, USA) was used for data analysis.

## Results

A total of 42 patients were included in the cohort (24 males and 18 females), with a mean age of 47.8 ± 10.8 years. Extracorporeal membrane oxygenation support was provided in one case, ventilator support in three, and oxygen masks in nine. None of the participants had a history of heart disease or chronic respiratory disease. The patients’ demographic characteristics, clinical features, and relevant medical history are shown in Table 1.

**Table 1:** Demographic characteristics, clinical features, and relevant medical history of the patients (*n* = 42)

Characteristics	Patients (42 cases)
Sex(M/F)	24 (18)
Age(y)	47.8 ± 10.8
Fever	36 (85.7%)
Cough	34 (80.9%)
Dyspnea	36 (85.7%)
Myalgia	37 (88.1%)
Diarrhea	16 (38.1%)
Heart disease history	0
Chronic lung disease history	0

LUS and pulmonary arterial pressure in all patients changed significantly as the disease progressed. The two parameters were positively correlated within three days of admission, and the correlation was stronger in patients with more severe disease (on days 3, 8, and 13: r = 0.448, *p* = 0.003; r = 0.738, *p* < 0.001; r = 0.325, *p* = 0.036, respectively; Fig. 1).

The LUS directly reflects the degree of lung disease, whereas the pulmonary arterial pressure is an indirect indicator. Increased pulmonary arterial pressure can directly cause dilation of the pulmonary artery trunk, with consequent enlargement of the right ventricle and right atrium, leading to the exacerbation of tricuspid regurgitation. One case of this occurrence is shown in Fig. 2, illustrating the relationship between pulmonary artery changes and tricuspid regurgitation on days 3, 8, and 13. There were significant differences in the LUS between the 3 days considered. Differences in the inner diameters of the atria, ventricles, and pulmonary artery are shown in Tables 2 and 3.

**Table 2:** Overall differences in LUS, pulmonary arterial pressure, and atrial diameter on days 3, 8, and 13

Category	On day 3	On day 8	On day 13	p
LUS score	6.1 ± 1.0	11. 1 ± 1.4	7.6 ± 2.1	0.000
PAP (mm Hg)	26.5 ± 5.7	42.6 ± 8.2	31.3 ± 6.4	0.000
PA (mm)	22.8 ± 1.6	24.6 ± 1.2	23.5 ± 1.4	0.000
RAV (ml)	35.1 ± 5.7	39.3 ± 5.9	37.3 ± 5.6	0.000
RVID (mm)	36.7 ± 2.2	39.0 ± 2.2	36.0 ± 1.7	0.000
RV-b (mm)	25.7 ± 1.9	29.6 ± 2.8	26.1 ± 1.8	0.000
LAV (ml)	35.5 ± 4.4	35.7 ± 3.3	35.4 ± 3.3	0.910
LVV (ml)	95.6 ± 13.9	97.5 ± 13.8	97.3 ± 13.8	0.761

**Table 3:** Differences in LUS, pulmonary artery pressure, and atrial diameter on days 3, 8, and 13

Day	LUS	PAP	PA	RAV	RVID	RV-b	LAV	LVV
	p	p	p	p	p	p	p	p
3/8	0.000	0.000	0.000	0.002	0.000	0.000	0.747	0.485
3/13	0.000	0.001	0.052	0.953	0.148	0.424	0.930	0.582
8/13	0.000	0.000	0.001	0.000	0.000	0.000	0.681	0.876

All patients exhibited an overall increase in both pulmonary arterial pressure and LUS on day 8; however, at that point in time there were four patients without any significant increase in the pulmonary arterial pressure and three without any significant increase in LUS. Comparisons of CT findings on day 8 revealed increased severity compared to day 3 in six patients, suggesting that these six patients were false negatives. In one (1/7) patient there was no substantial difference between the CT findings acquired on day 3 and those acquired on day 8, indicating a negative result in this case. There were 41/42(97.6%) cases in which the two in combination were positive (Table 4).

**Table 4:** Positivity rates of LUS and pulmonary arterial pressure values, considered alone or in combination

	PAP	LUS	PAP + LUS
Positive	39 (92.9%)	38 (90.5%)	41 (97.6%)
Negative	3 (7.1%)	4 (9.5%)	1 (2.4%)
Total	42	42	42

Note -Positive: the pulmonary arterial pressure was greater than 30 mmHg in 3 days; LUS greater than 1

## Discussion

In the present study, the LUS was positively correlated with pulmonary arterial pressure, with the correlation becoming stronger as the disease severity worsens. Increased pulmonary arterial pressure may be caused by several pathologies [15, 18, 19]. Patients with a history of underlying cardiopulmonary disease were excluded from the present study; the changes observed in the pulmonary arterial pressure were assumed to be related to the current lung disease. During lung inflammation, inflammatory infiltration and alveolar exudate reduce the alveolar surface area available for diffusion, thereby prolonging the diffusion time. Hypoxic acidosis may cause pulmonary endothelial edema and vasospasm, leading to PH [15, 20].

COVID-19 pneumonia and the 2003 SARS outbreaks were both caused by members of the coronavirus family. Angiotensin-converting enzyme-2 is a component of the renin-angiotensin system that protects blood vessels and possibly a functional receptor for coronaviruses on epithelial cells [21–23]. Another component of the renin-angiotensin system is angiotensin II, which causes inflammation and damage of the alveolar epithelium. The lung alterations associated with COVID-19 may be due to the upregulation of angiotensin II and reduced angiotensin-converting enzyme-2 levels, resulting in increased pulmonary vasoconstriction. The results of the present study suggest that these factors may be associated with higher LUSs and higher pulmonary arterial pressure.

The assessment of pulmonary artery pressure can be performed using several methodologies. Right heart catheterization is an advanced and reliable method for this task; however, it is performed only in specific cases due to its invasive nature [16, 24]. Abbas et al. developed an algorithm to calculate the PVR of patients with pre occipital PH; however, this method may lead to false-positive results [25]. The most commonly used method in clinical practice is the calculation of maximum pulmonary systolic pressure [17, 26, 27] using the tricuspid regurgitation velocity [28]. This method has been widely used and is based on the clear guidelines of the ESC and ERS. Therefore, we opted to use these guidelines to evaluate the pulmonary artery pressure in our study; however, this method requires good image quality and sufficient Doppler signal. These characteristics may not always be present, though all the images of the patients selected for the study met the requirements.

Several studies [15–19] investigated pulmonary arterial hypertension caused by pneumonia; however, there are no reports on the use of pulmonary arterial pressure to predict the LUS. In the present study, the LUS was dynamically evaluated, and echocardiography was performed simultaneously. The LUS was positively correlated with disease severity. Echocardiography indicated that the amount of tricuspid regurgitation increased with aggravating disease conditions; the pulmonary arterial pressure and the inner diameters and volumes of the pulmonary artery, right ventricle, and right atrium also increased. All these parameters exhibited statistically significant changes during disease progression, and all of them decreased with improving conditions (Tables 2 and 3). These results suggest that changes in LUS and pulmonary arterial pressure can reflect the severity of lung lesions.

Hemodynamic principles indicate that increased pulmonary arterial pressure leads to increased right ventricular ejection resistance and, consequently, increased inner diameters of the right atrium, right ventricle, and pulmonary artery. Dynamic echocardiography can be used to monitor changes in pulmonary arterial pressure and the size of each heart chamber in real time; therefore, the disease progression can be assessed efficiently. The positive correlation between pulmonary arterial pressure and LUS in the current study also indicates a tendency for LUS. There were no significant changes in left heart size during the entire course of the disease, indicating a low probability of left heart involvement, consistent with previous studies.

In the present study, the positivity rate for increased pulmonary arterial pressure on day 8 was 92.9%. Three patients (7.1%) had stable pulmonary arterial pressure, possibly indicating compensation by the pulmonary blood vessels [13, 20]. The positivity rate for LUS increase on day 8 was 90.5%, though the scores of four patients (9.5%) did not increase with aggravating disease conditions. In conjunction with CT findings, this result suggests that the regions of lesion exacerbation in these cases were located in the innermost pulmonary areas, beyond ultrasound detection range, which are consistent with the principles of lung ultrasound. The positivity rate for the two parameters combined was 97.6%, improving the accuracy of disease progression evaluation. Currently, no reports have examined this aspect.

The current study had some limitations. Some patients required oxygen therapy for dyspnea; compared with patients who did not require such, the estimation of pulmonary arterial pressure may be biased. Moreover, since the frequency of CT examinations was lower than that of ultrasound, not all patients have corresponding CT data at the three time-points considered, causing a lack of basis for comparison. However, the corresponding CT data were available for the seven negative patients for either parameter, providing a reliable reference for diagnosis. Some previous studies [8, 9] describe the use of LUS and echocardiography in patients with COVID-19 pneumonia. On the other hand, cardiac reports [9] focus on intensive care unit (ICU) patients, while most of the patients we evaluated were non-ICU patients. Our comprehensive pulmonary evaluation (bilateral LUS) may reflect the disease progression more accurately, as the change in pulmonary hemodynamics in patients with COVID-19 pneumonia is evident. Moreover, we found the modification in pulmonary arterial pressure to be a sensitive indicator at the early stage of the disease. In patients on ventilator support, when it is challenging to obtain double lung scoring, the pulmonary arterial pressure can be measured to assess the severity of lung disease. This knowledge may help clinicians in the management of patients with severe disease.

## Conclusions

In COVID-19 pneumonia patients, the pulmonary arterial pressure and LUS are positively correlated. For those who are unable to be transferred or relocated, the pulmonary arterial pressure may be measured to reflect the degree of lung lesions indirectly. Combining these two parameters could improve the accuracy of lung disease progression assessment, providing an important guiding role for treatment decisions.

## Data Availability

The datasets generated and/or analysed during the current study are not publicly available due [REASON WHY DATA ARE NOT PUBLIC] but are available from the corresponding author on reasonable request.

## Abbreviations

COVID-19: Coronavirus disease 2019
CT: Computed tomography
LUS: Lung ultrasound score
RT-PCR: Reverse transcriptase polymerase chain reaction
